# Inhibitor of DNA binding 1 as a secreted angiogenic transcription factor in rheumatoid arthritis

**DOI:** 10.1186/ar4507

**Published:** 2014-03-13

**Authors:** Takeo Isozaki, M Asif Amin, Ali S Arbab, Alisa E Koch, Christine M Ha, Gautam Edhayan, G Kenneth Haines, Jeffrey H Ruth

**Affiliations:** 1 University of Michigan Medical School, Ann Arbor, MI 48109, USA; 2 Henry Ford Hospital & Medical Centers, Detroit, MI 48202, USA; 3 Veterans Administration Healthcare System, Ann Arbor, MI 48109, USA; 4 Yale University School of Medicine, New Haven, CT 06520, USA; 5 Department of Medicine, Division of Rheumatology, University of Michigan Medical School, 109 Zina Pitcher Drive, 4023 BSRB, Ann Arbor, MI 48109-2200, USA

## Abstract

**Introduction:**

Rheumatoid arthritis (RA) is characterized by enhanced blood vessel development in joint synovium. This involves the recruitment of endothelial progenitor cells (EPCs), allowing for *de novo* vessel formation and pro-inflammatory cell infiltration. Inhibitor of DNA Binding 1 (Id1) is a transcription factor characteristic of EPCs that influences cell maturation.

**Method:**

Enzyme-linked immunosorbant assay (ELISA) and polymerase chain reaction (PCR) were used to examine Id1 levels in synovial fluid (SF) and endothelial cells (ECs), respectively. Immunohistology was used to determine the expression of Id1 in synovial tissue (ST). Human dermal microvascular EC (HMVEC) migration and tube forming assays were used to determine if recombinant human Id1 (rhuId1) and/or RA SF immunodepleted Id1 showed angiogenic activity. We also utilized the RA ST severe combined immunodeficient (SCID) mouse chimera to examine if Id1 recruits EPCs to RA synovium.

**Results:**

ST samples immunostained for Id1 showed heightened expression in RA compared to osteoarthritis (OA) and normal (NL) ST. By immunofluorescence staining, we found significantly more Id1 in RA compared to OA and NL vasculature, showing that Id1 expressing cells, and therefore EPCs, are most active in vascular remodeling in the RA synovium. We also detected significantly more Id1 in RA compared to OA and other arthritis SFs by ELISA, which correlates highly with Chemokine (C-X-C motif) ligand 16 (CXCL16) levels. *In vitro* chemotaxis assays showed that Id1 is highly chemotactic for HMVECs and can be attenuated by inhibition of Nuclear Factor κB and phosphoinositide 3-kinase. Using *in vitro* Matrigel assays, we found that HMVECs form tubes in response to rhuId1 and that Id1 immunodepleted from RA SF profoundly decreases tube formation in Matrigel *in vitro*. PCR showed that Id1 mRNA could be up-regulated in EPCs compared to HMVECs in response to CXCL16. Finally, using the K/BxN serum induced arthritis model, we found that EC CXCR6 correlated with Id1 expression by immunohistochemistry.

**Conclusions:**

We conclude that Id1 correlates highly with CXCL16 expression, EPC recruitment, and blood vessel formation in the RA joint, and that Id1 is potently angiogenic and can be up-regulated in EPCs by CXCL16.

## Introduction

RA is a debilitating inflammatory joint disease in which microvascular expansion in the joint lining is a characteristic finding. Synovial neovascularization occurs pre-symptomatically and is critical for disease progression [1]. Expansion of the microcirculation requires either the proliferation of existent vascular endothelial cells (ECs) (angiogenesis), or the recruitment from the bone marrow (BM) of endothelial progenitor cells (EPCs) (vasculogenesis) [2]. Recruitment is orchestrated by vessel lumen expression of adhesion molecules that capture circulating EPCs [3], and of chemokines that direct EPC migration into surrounding tissues [4]. Over the past decade, EPCs have emerged as important regulators of cardiovascular integrity [5]. However, the specific molecular mechanisms that mediate EPC recruitment remain poorly understood. Additionally, little information exists regarding the relative contribution of EPCs to the synovial neovascularization that occurs in RA.

EPCs have been identified both in the human mononuclear leukocyte fraction of peripheral blood (PB) [6], and in their site of origin, the BM [7]. EPCs can be detected in the PB, expressing a variety of cell surface markers, which identify them as vascular and BM derived. Key EPC markers include vascular endothelial (VE) cadherin, vascular endothelial growth factor receptor 2 (VEGFR2), CD31^low^, c-kit and prominin 1/AC133 [8-11]. However, the expression of these markers differs depending on whether the EPCs are in the BM, PB or in tissues such as tumors or the RA synovium.

Previous studies have shown EPCs uniquely express the transcription factor Id1. Id1 is a member of the helix-loop-helix (HLH) family of transcription factors and a marker of self-renewal [12]. Inhibition of Id1 in the BM results in significant EPC linked tumor vascular defects [9,13]. This strongly suggests that Id1 is a true marker of EPCs. To this end, the need exists to clearly identify EPCs in RA tissues, and to better characterize what exactly governs their recruitment. CXCL16 is a chemokine known to be very highly expressed in RA tissues [14-16]. Interestingly, human and murine EPCs have also been shown to express the CXCL16 receptor CXCR6, suggesting that this ligand-receptor pair might be a principal factor for EPC recruitment into the RA joint [17].

We have evidence that EPCs utilize the CXCL16-CXCR6 ligand-receptor pair for recruitment purposes, and are associated with Id1 expression in RA. Considering the known function of the CXCR6 receptor in relation to recruitment and homing of immune cells in RA [14], it is reasonable to expect that CXCR6 may also be involved in the recruitment and homing of Id1 expressing mesenchymal stem cells (MSCs) to RA synovium, likely for the purposes of tissue regeneration and/or vasculogenesis [17]. We show that Id1 is intrinsic to this process and together with the CXCL16-CXCR6 ligand-receptor pair, work to bring EPCs from the BM to the RA joint.

## Methods

### Rodents

Animal care at the Unit for Laboratory Animal Medicine (ULAM) at the University of Michigan is supervised by a veterinarian and operates in accordance with federal regulations. Severe combined immunodeficient (SCID) and C57BL/6 mice were obtained from the National Cancer Institute (NCI). All efforts were made to reduce stress or discomfort to all animals. All rodents were given food and water *ad libidum* throughout the entire study and were housed in sterile rodent micro-isolator caging with filtered cage tops in a specific pathogen-free environment to prevent infection. Approval to use animals for all parts of this study was obtained from the ethics committee at the University of Michigan Committee on the Use and Care of Animals (UCUCA).

### K/BxN serum-induced arthritis model

K/BxN breeder mice were provided by Drs. Mathis and Benoit. To generate arthritic K/BxN mice, K/B positive mice were crossed with NOD/LTj mice as previously described [18]. Naïve wild type (Wt) and CXCR6 gene knockout (CXCR6^−/−^) mice at the age of five to seven weeks were injected with 150 μl of K/BxN serum *i.p.*, and this was considered to be Day 0 of arthritis. Another injection of 150 μl of K/BxN serum followed on Day 2. Robust arthritis with severe swelling of the joints typically developed on Day 5. Mouse ankles were harvested for histology typically by Day 12.

### Isolation of EPC CD34^+^ cells from cord blood

Human EPCs were isolated from cord blood from granulocyte-colony stimulating factor (G-CSF) mobilized leukopheresis samples on the basis of CD133 expression, using an antibody coupled magnetic bead cell isolation system (Stem Cell Technologies, Vancouver, BC, Canada). Human umbilical cord blood was collected by the method of Moore *et al.*[19] as previously described [20]. To confirm purity of the EPCs, isolated cell populations were subjected to flow cytometry analysis as described previously [21,22]. EPCs with appropriate cell markers (CD34^+^, CD133^+^, CD14^−^) were used in chimeras and related *in vitro* studies.

### Neutralization of Id1 in RA SFs

RA SFs were pre-incubated either with mouse anti-human Id1 antibody (Abcam, Cambridge, MA, USA) or with an equivalent amount of a corresponding control antibody (non-specific mouse IgG) for two hours at 4°C. Samples were mixed with Protein A/G agarose (Millipore, Billerica, MA, USA), and rotated overnight at 4°C. Samples were centrifuged briefly to pellet the Id1/antibody/Protein A/G complex and the Id1 depleted SFs were collected.

### ELISA for Id1 and CXCL16

Rheumatoid factor (RF) was depleted from human SFs using anti-human IgM (μ-chain specific) agarose antibody (Sigma-Aldrich, St. Louis, MO, USA). Levels of Id1 were measured using 96-well plates. RA, OA and other disease SFs, and Id1 as a standard were coated in duplicate for one hour. The plates were washed with wash buffer and coated with blocking buffer. Mouse anti-human Id1 antibody (Abcam) in blocking buffer was added for one hour. Subsequently, biotinylated goat anti-mouse antibody (Vector Labs, Burlingame, CA, USA) and streptavidin-HRP (BD Biosciences, San Jose, CA, USA) were added, and the concentration in samples was measured at 450 nm after developing the reaction with tetramethylbenzine substrate (TMB, Sigma-Aldrich). For the CXCL16 ELISA, 96-well plates were coated with rabbit anti-human CXCL16 (PeproTech, Rocky Hill, NJ, USA). SFs and rhuCXCL16 (PeproTech) as a standard were added. Biotinylated rabbit anti-human CXCL16 antibody (PeproTech) was used to detect CXCL16 using a streptavidin-HRP, with TMB. The concentration in each sample was measured at 450 nm.

### Immunohistologic analysis

Tissue slides were fixed in cold acetone for 20 minutes. Following incubation with 3% H_2_O_2_ for five minutes to block endogenous peroxidase, STs were blocked with 20% fetal bovine serum (FBS) and 5% goat serum in phosphate-buffered saline (PBS) at 37°C for one hour, and then incubated with mouse anti-human Id1 antibody (Abcam, 10 μg/ml), rabbit anti-mouse Id1 antibody (Cal Bioreagents, San Mateo, CA, USA) or purified nonspecific IgG for one hour at 37°C in blocking buffer. The ST samples were washed with PBS, and a 1:200 dilution in blocking buffer of biotinylated goat anti-mouse or anti-rabbit antibody was added and incubated for an additional 30 minutes at 37°C. After washing, antibody binding was detected using a Vectastain ABC Elite kit (Vector Labs) and the chromogen 3,3′-diaminobenzidine (DAB) (Vector Labs). ST samples were counterstained with Harris hematoxylin. Staining was evaluated by a pathologist who was blinded with regard to the sample group. Slides were examined for cellular immunoreactivity, and cell types were distinguished based on their characteristic morphology. The percentage of cells expressing Id1 was analyzed and graphed.

### Immunofluorescence (IF)

The slides were fixed in cold acetone for 30 minutes. The STs were blocked with 5% donkey serum and 20% FBS in PBS at 37°C for one hour, and then incubated with mouse anti-human Id1 antibody (Abcam, 10 μg/ml) and rabbit anti-human von Willebrand factor (vWF) antibody (DAKO, Carpinteria, CA, USA), or purified nonspecific mouse and rabbit IgG for one hour at 37°C in blocking buffer. The ST samples were washed with PBS, and a 1:200 dilution in blocking buffer of fluorescent conjugated donkey anti-mouse and donkey anti-rabbit antibody was added and incubated for an additional one hour at 37°C.

### RNA extraction and quantitative RT-PCR

Total RNA was isolated from HMVECs and EPCs using RNAeasy mini RNA isolation kits in conjunction with QIAshredders (Qiagen, Valencia, CA, USA) following the manufacturer’s protocol. Following isolation, RNA was quantified and checked for purity using a spectrophotometer (Nanodrop Technologies, Wilmington, DE, USA). cDNA was then prepared using a Verso cDNA kit (Thermo Fisher Scientific) as per the manufacturer’s protocol. Quantitative PCR (qPCR) was performed using Platinum SYBR Green qPCR SuperMix-UDG (Life Technologies, Carlsbad, CA, USA) following the manufacturer’s protocol. The primer pairs used were based on published sequences. Diluted cDNA was mixed with Platinum SYBR green qPCR SuperMix-UDG, forward and reverse primers specific for each gene (10 μM final concentrations), and incubated at the following cycles; 50°C for 2 minutes, 95°C for 2 minutes and 40 cycles of 95°C for 30 sec, 55°C for 30 sec and 68°C for 30 sec using an ABI Prism 7500 sequence detection system (Applied Biosystems). The primers for human Id1 [23], are forward: AGAACCGCAAGGTGAGCAA and reverse: CCAACTGAAGGTCCCTGATGTAG. The primers used for β-actin were the same as we used previously [24], and are forward: GCTAGGCAGCTCGTAGCTCT and reverse: GCCATGTACGTTGCTATCCA. All samples were run in duplicate.

### HMVEC chemotaxis to Id1

HMVECs (BioWhittaker, Walkersville, MD, USA) were maintained in growth factor complete endothelial basal media (EBM) supplemented with 5% FBS. Cells were between passages 7 and 10, and did not display discernable phenotypic changes when observed before each assay. Cells were maintained at 37°C and 5% CO_2_. HMVEC migration *in vitro* was tested using a modified 48-well Boyden chemotaxis chamber (Neuroprobe, Cabin John, MD, USA). HMVECs (1 × 10^6^ cells/ml EBM + 0.1% FBS) were plated in the bottom wells of the chambers with a polyvinylpyrolidone-free polycarbonate filter (8-μm pore size; Nucleopore, Pleasant, CA, USA). The chambers were inverted and incubated in a humidified incubator with 5% CO_2_/95% air at 37°C for two hours, allowing HMVECs to attach to the membrane. The chambers were inverted again and Id1 was added at different concentrations, with PBS, or basic fibroblast growth factor (bFGF, 60 nM) used as negative and positive controls, respectively. After incubation for two hours at 37°C, the membranes were removed, fixed in methanol for one minute, and stained with Diff-Quick (VWR International, West Chester, PA, USA). Cell migration was determined in quadruplicate and analyzed in three high-power 40X fields per well. The experiment was performed four times. Data are expressed as the number of cells migrating per well.

### Signal inhibited chemotaxis assay

To determine which kinases were required for Id1 mediated HMVEC chemotaxis, cells were incubated with chemical signaling inhibitors. HMVECs were pre-incubated with chemical signaling inhibitors for one hour prior to the assay, and the inhibitors were present in the lower chamber with the HMVECs during the assay. The following inhibitors were purchased from and used at concentrations recommended by Calbiochem (La Jolla, CA, USA): PD98059 (10 μM Erk1/2 inhibitor), PDTC (100 μM nuclear factor kappa B (NFκB) inhibitor), LY294002 (10 μM PI3K inhibitor), SB203580 (10 μM p38 MAPK inhibitor) and PP2 (1 μM Src inhibitor).

### Matrigel tube formation assay

Matrigel tube formation assay using growth factor-reduced Matrigel (BD Biosciences) was performed. HMVECs were seeded in Labtek chamber slides on growth factor reduced Matrigel (Becton Dickinson, Bedford, MA, USA) at a density of 1.6 × 10^4^ cells per chamber. The test substances used were rhuId1 (1 and 10 nM), bFGF (60 nM, R&D Systems, Minneapolis, MN, USA; positive control) and PBS (negative control). The treated HMVECs (1.8 × 10^4^ cells/400 μl) were plated on Matrigel in the presence of Id1, bFGF or PBS for six hours at 37°C. Photographs (100×) were taken and tubes were counted by a blinded observer. Tubes were defined as elongated connecting branches between two identifiable HMVECs. SFs were diluted 1:100 with PBS. Matrigel tube formation assay was performed using SFs and PBS. Photographs (100×) were taken and tubes were counted by a blinded observer.

### RA ST SCID mouse chimera

The backs of six- to eight-week-old SCID mice (NIH/NCr) were shaved and graft beds prepared (1 × 1 × 0.05 cm). A single graft was implanted per animal (approximately 0.5 cc). Human RA STs were implanted and transplants sutured while mice were anesthetized. Grafts were allowed to “take” and used at approximately four weeks after surgery when animals did not experience gross evidence of inflammation other than the anticipated neovascularization. Once grafts took, 2.5 **×** 10^5^ fluorescently dye-tagged (PKH26 dye, Sigma-Aldrich, St. Louis, MO, USA) EPCs were injected *i.v.* into mice while they were receiving simultaneous intragraft injections of RA SF (100 μl) that was either “sham” immunoneutralized with non-specific IgG or immunoneutralized with the specific antibody to human Id1. For some experiments, RA ST SCID mouse chimeras were injected with 2.5 **×** 10^5^ fluorescently dye-tagged human EPCs while receiving simultaneous injections of either human Id1 (10 nM) or PBS (100 μl). EPCs were allowed to circulate for 72 hours. Grafts were then harvested, cryosectioned and examined using a fluorescence microscope. Human RA ST grafts as well as murine organs, such as lymph nodes (LNs), spleen, kidney, heart, lung, liver and brain, were harvested at the time of sacrifice. These tissues were examined for non-specific EPC recruitment to non-targeted tissues to ensure that adoptively transferred EPCs were recruited only to the engrafted synovium. All engrafted STs, as well as various organs, were snap-frozen in liquid nitrogen, and stored at −80°C until further processing.

### Statistical analysis

Results are expressed as the mean ± standard error of the mean (SEM). Data were analyzed using a Student’s *t*-test. *P*-values less than 0.05 were considered significant.

## Results

### ELISA for Id1 and CXCL16 on SFs

**Figure 1:**
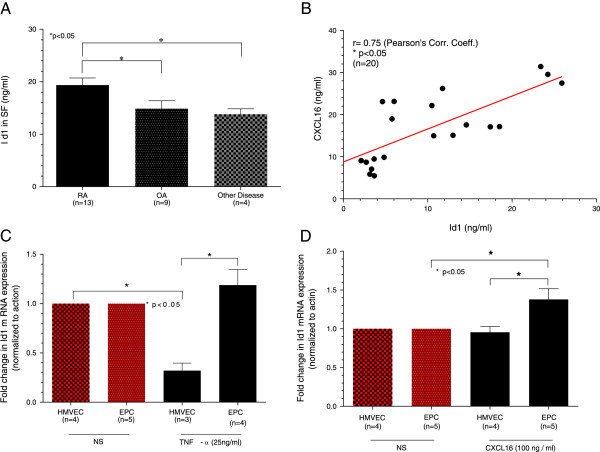
**Id1 and CXCL16 are linked in RA SF and at the level of transcription in EPCs.** An ELISA was developed to detect Id1 in synovial fluids (SFs) taken from rheumatoid arthritis (RA), osteoarthritis (OA) and patients with other arthritic disorders, including gout, seronegative spondyloarthritis and systemic lupus erythematosus. **A)** The ELISA showed that Id1 was found in SFs of other diseases (n = the number of patients = 4, 13.8 (mean) ± 0.9 (standard error of mean (SEM)) ng/ml) and was significantly higher in RA SFs (n = 13, 19.4 ± 1.3 ng/ml), and OA SF (n = 9, 14.9 ± 1.4 ng/ml), all of which were *P* <0.05 (n = number of patients). **B)** CXCL16 was measured in the same samples as Id1, and correlated with Id1 expression in RA SF (r = 0.75 Pearson’s Correlation Coefficient (*P* <0.05, n = number of patients). **C)** Total RNA was isolated from human dermal microvascular endothelial cells (HMVECs) and endothelial progenitor cells (EPCs). Following isolation, RNA was quantified and checked for purity using a spectrophotometer and made into cDNA and amplified in a thermocycler (40 cycles). As shown, tumor necrosis factor-alpha (TNF-α) did not affect Id1 mRNA levels in EPCs, and reduced the number of Id1 transcripts in HMVECs. **D)** CXCL16 stimulation elevated Id1 mRNA expression in EPCs but not HMVECs, indicating that CXCL16 and Id1 are associated at the level of transcription in EPCs but not mature ECs (n = number of independent experimental replicates).

Id1 is expressed and secreted in SFs, and can be measured in RA, OA and other disease SFs (two gout, one seronegative spondylitis, and one lupus SF). As shown, Id1 is elevated in RA compared to OA and other disease SFs (Figure 1A), taken from a patient population around the same point in time to ensure that we controlled for any possible effects on Id1 and CXCL16 concentration measurements from the storage conditions. Similarly for CXCL16, 96-well plates were coated with rabbit anti-human CXCL16 (PeproTech). The identical RA SFs were used for Id1 and CXCL16 measurements for the correlation studies. We found that soluble Id1 highly correlates with CXCL16 in RA SF (Figure 1B), indicating that CXCL16 and EPC migration are linked in RA SF (Pearson’s correlation coefficient r = 0.75; n = 20).

### mRNA extraction and quantitative RT-PCR

Total RNA was isolated from stimulated (tumor necrosis factor-alpha (TNF-α) or CXCL16) or non-stimulated HMVECs and EPCs. The data are presented as fold increases compared to non-stimulated (NS) ECs that serve as the control. TNF-α did not affect Id1 mRNA in EPCs, but significantly reduced the number of Id1 transcripts in HMVECs compared to NS HMVECs (Figure 1C). In addition, there was a significant reduction of Id1 transcripts between HMVECs and EPCs stimulated with TNF-α. We also found significantly elevated Id1 mRNA expression in EPCs compared to HMVECs when cells were stimulated with CXCL16, and that CXCL16 up-regulates Id1 expression in EPCs, but not HMVECs, indicating that CXCL16 and Id1 are associated at the level of transcription in EPCs (Figure 1D).

### Histology for Id1 was performed on RA, OA and NL ST sections

**Figure 2:**
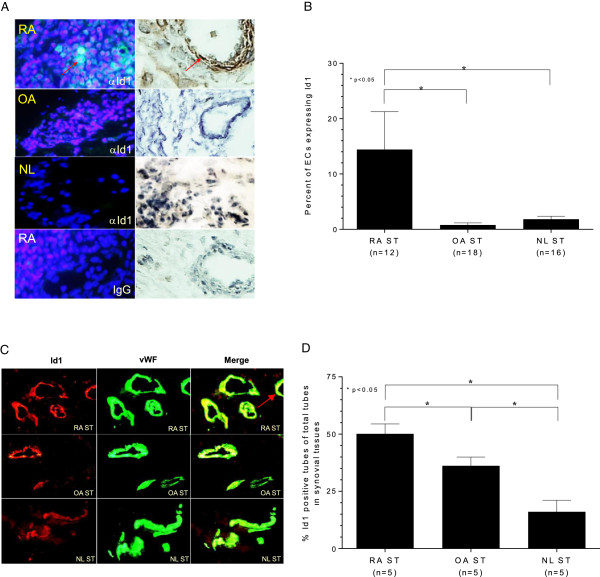
**IF was performed on RA, OA, and NL ST sections: A) For IF histology, samples were incubated with mouse anti-human Id1 antibody then followed with a fluorescent conjugated secondary antibody.** Samples were stained with DAPI (blue) and images merged. Id1 expression is clearly shown in green (images were taken at 400×). IHC was also performed on RA, OA, and NL ST sections as described. As shown, Id1 could be seen up-regulated in RA vascular tissue by IF staining and IHC. **B)** The percentage of cells expressing Id1 were analyzed on ECs with a significantly higher percentage of Id1 expressing ECs in RA compared to NL and OA ST (n = number of patients). **C)** For merged IF histology, samples were incubated with mouse anti-human Id1 and rabbit anti-human vWF antibodies. This was followed by fluorescent conjugated secondary antibodies and images were merged. Id1 expression is clearly shown in red and vessels as defined by vWF expression are shown in green (images taken at 400×). Merged vessel regions shown in yellow are due to Id1 expressing EPCs. **D)** The percentages of Id1 positive tubes in each of the tissues were calculated and graphed. Id1 containing vasculature was significantly higher in RA compared to NL and OA ST (400×, n = number of patients).

Id1 is highly expressed in the vasculature of RA ST, but less so in OA or NL ST, suggesting that the micro-environment of the RA joint either facilitates Id1 expression or is favorable to EPC migration (Figure 2A). The results are graphed and show that Id1 is clearly present on a higher percentage of ECs in RA compared to OA and NL ST (Figure 2B). Id1 and vWF can be seen in all tissues, but the highest amounts of both vasculature and Id1 expression can be seen in RA compared to OA and NL ST (shown embedded in yellow; see arrow; Figure 2C). Images were taken at 400× and merged. The percentage of Id1 positive tubes was calculated and expressed in the graph. Significantly higher percentages of Id1 expressing tubes were identified in RA compared to OA and NL ST, indicating that vasculogenesis due to EPC migration to synovium is elevated in RA synovium (Figure 2D).

### HMVEC chemotaxis assay

**Figure 3:**
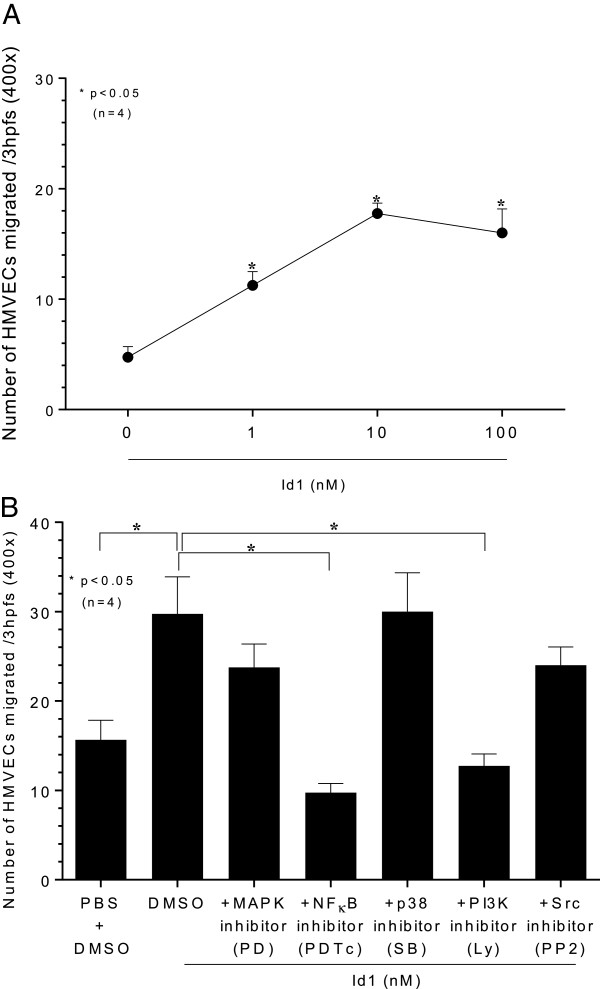
**Chemotaxis: to determine the kinases required for Id1 mediated HMVEC chemotaxis, cells were incubated with chemical signaling inhibitors.** Human dermal microvascular endothelial cells (HMVECs) were pre-incubated with chemical signaling inhibitors for one hour prior to the assay, and the inhibitors were present in the lower chamber with the HMVECs during the assay. The following inhibitors were purchased from and used at concentrations recommended by Calbiochem (La Jolla, CA): PD98059 (10 μM Erk1/2 inhibitor), PDTc (100 μM NFκB inhibitor), LY294002 (10 μM PI_3_K inhibitor), SB203580 (10 μM p38 MAPK inhibitor) and PP2 (1 μM Src inhibitor). HMVEC migration assays were performed by placing cells in a 48-well neuroprobe microchemotaxis chamber. **A)** HMVECs were chemotactic for Id1 at physiologically relevant concentrations of Id1 (1, 10 and 100 nM, n = number of independent experimental replicates). **B)** We examined HMVEC signaling pathways to Id1 using signaling inhibitors, and performed HMVEC chemotaxis assays at the peak concentration of Id1 chemotactic activity (10 nM). We found that PDTc (NFκB inhibitor) and Ly (PI_3_K inhibitor) significantly reduced HMVEC migration towards Id1. The other inhibitors used had no effect upon Id1 HMVEC chemotaxis (n = number of independent experimental replicates).

HMVEC chemotaxis assays to rhuId1 (1 to 100 nM) were performed. Readings represent the number of cells migrating through the membrane (the sum of three high-power fields (400×)/well, averaged for each quadruplicate well). Id1 displayed potent chemotactic activity for HMVECs at the three doses tested (1, 10 and 100 nM), but was most active at 10 nM (Figure 3A). We examined HMVEC signaling pathways in response to Id1 using signaling inhibitors and performed HMVEC chemotaxis assays at the peak concentration of Id1 chemotactic activity (10 nM). We found that PDTC (NFκB inhibitor) and Ly (PI_3_K inhibitor) significantly reduced HMVEC migration towards Id1. The other inhibitors used (PD, MAPK inhibitor; SB, p38 inhibitor; PP2, Src inhibitor) had no effect upon Id1 HMVEC chemotaxis (Figure 3B).

### Capillary morphogenesis assay shows that Id1 is angiogenic

**Figure 4:**
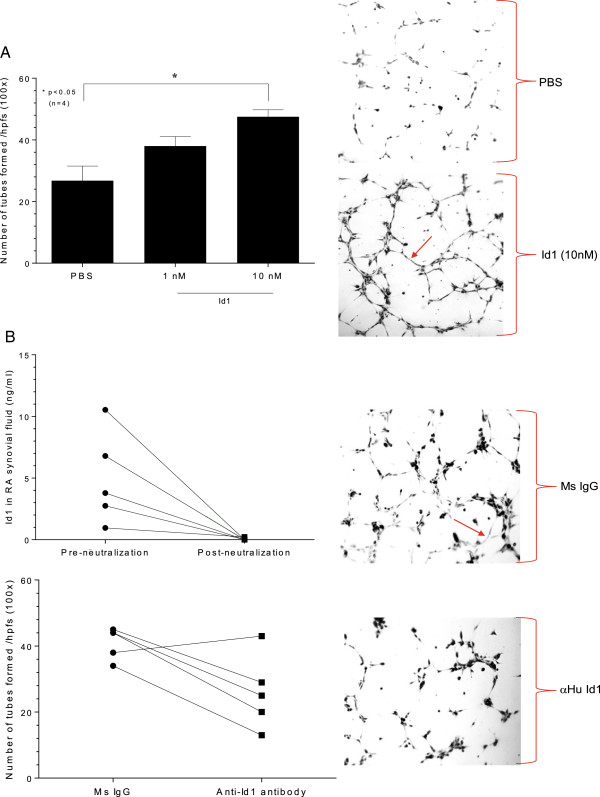
***In vitro *Matrigel tube formation assay. A)** Matrigel tube formation assay using growth factor-reduced Matrigel (BD Biosciences) was performed. The test substances used were recombinant human Id1 (rhuId1) (1 and 10 nM), basic fibroblast growth factor (bFGF) (60 nM, R&D Systems, positive control) and phosphate-buffered saline (PBS) (negative control). The treated human dermal microvascular endothelial cells (HMVECs) (1.8 × 10^4^ cells/400 μl) were plated on Matrigel in the presence of Id1, bFGF or PBS for six hours at 37°C. Photographs (100×) were taken and tubes were counted by a blinded observer. Tubes were defined as elongated connecting branches between two identifiable HMVECs. A) HMVECs formed tubes to Id1 at 10 nM (the concentration for peak HMVEC chemotactic activity for HMVECs, n = number of independent experimental replicates). **B)** Rheumatoid arthritis (RA) synovial fluid (SF) was pre-incubated with mouse anti-human Id1 antibody (Abcam) for two hours at 4°C. RA SFs were added in the Protein A agarose beads and were rotated at 4°C overnight. The next day, the samples were spun down and SFs collected. SFs were diluted 1:100 with PBS. We then measured Id1 in the SFs pre- and post-Id1 neutralization, and as shown, effectively eliminated Id1 from the SFs. RA SF depleted of Id1 showed less HMVEC tube forming activity compared to “sham”, IgG depleted SFs. Photographs (100×) were taken and tubes were counted by a blinded observer (n = number of independent experimental replicates).

HMVECs formed tubes to Id1 at 10 nM, which was the peak concentration for HMVEC chemotactic activity (Figure 4A). We then measured Id1 in the SFs pre- and post-Id1 neutralization, and as shown, anti-Id1 antibody effectively neutralized Id1 activity in the SFs. RA SF depleted of Id1 showed less HMVEC tube forming activity compared to “sham”, IgG depleted SFs. Photographs (100×) were taken and tubes were counted by a blinded observer (Figure 4B).

### EPCs migrate to Id1 in the RA ST SCID mouse chimera

**Figure 5:**
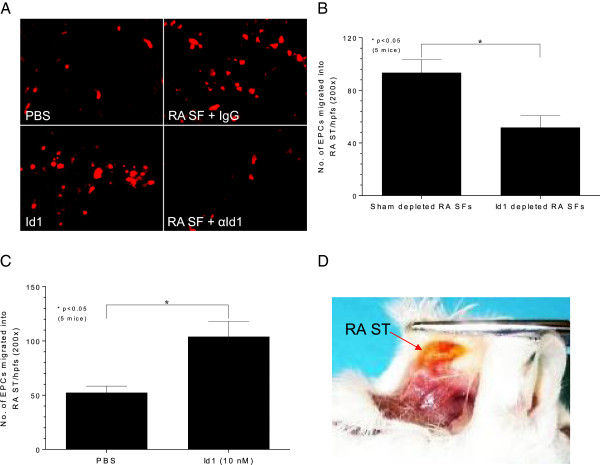
**RA ST SCID mouse chimera.** After a four-week engraftment period, mice were injected *i.v.* with 2.5 × 10^5^ fluorescently dye-tagged human endothelial progenitor cells (EPCs) while receiving simultaneous intragraft injections of rheumatoid arthritis (RA) synovial fluid (SF) (100 μl) that was either “sham” immunoneutralized with non-specific IgG or immunoneutralized with specific antibody to human Id1. In some experiments, RA synovial tissue (ST) SCID chimeric mice received dye-tagged EPCs while receiving simultaneous intragraft injections of human Id1 (10 nM) or PBS. **A)** Section from a SCID mouse injected with Id1 or sham immunodepleted RA SF showing significantly elevated EPC migration to the engrafted RA ST injected with “sham” immunodepleted RA SF. **B)** Approximately 50% fewer EPCs migrated to engrafted RA ST injected with Id1 neutralized from RA SF compared to sham injected RA SF (n = 10 sections from five mice (that is, two sections/mouse)). **C)** Less than 50% fewer EPCs migrated to engrafted RA ST in chimeric mice when injected intragraft with phosphate-buffered saline (PBS) compared to Id1 (n = 10 sections from five mice (that is, two sections/mouse)). **D)** Engrafted RA ST in the SCID mouse chimera.

Fluorescently dye-tagged EPCs were administered *i.v.* into mice receiving simultaneous intragraft injections of RA SF that was either “sham” immunoneutralized with non-specific IgG or immunoneutralized with specific antibody to human Id1. Approximately 50% fewer EPCs migrated to engrafted RA ST injected with RA SF depleted of Id1 compared to sham depleted injected RA SF (Figures 5A, B). RA ST SCID chimeric mice injected intragraft with Id1 (10 nM) compared to PBS had significantly elevated EPC migration to the engrafted RA ST, showing less than 50% fewer EPCs migrating to engrafted RA ST injected with PBS alone (Figure 5C). Also shown is a picture of engrafted RA ST in the SCID mouse chimera showing a viable RA ST graph (Figure 5D).

### Id1 expression is elevated in Wt, but not CXCR6^−/−^ K/BxN serum-induced mice

**Figure 6:**
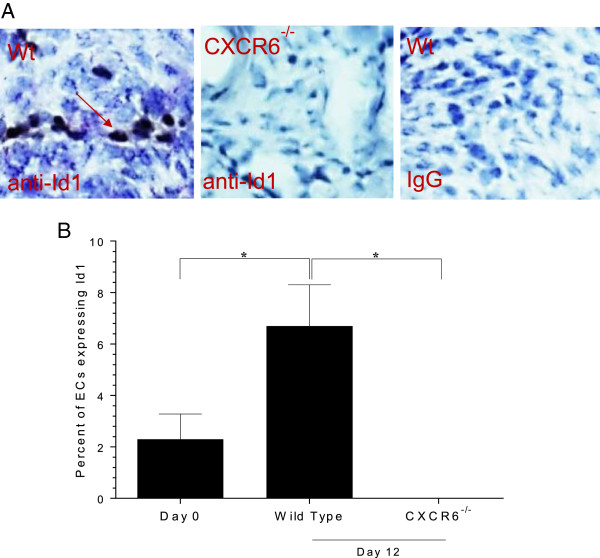
**Id1 expression is elevated in Wt, but not CXCR6**^**−/− **^**K/BxN serum-induced mice.** Wild type (Wt) and CXCR6^−/−^ mice were induced with K/BxN serum and joint tissue sections were immunostained for Id1. **A)** Day 12 Wt mice show clear expression of Id1 positive endothelial cells (ECs), whereas CXCR6^−/−^ mice do not. **B)** The results are graphed and show that day 0 and 12 Wt mice have Id1 expressing endothelial progenitor cells (EPCs) in joint tissue, but Id1 positive cells were not detected in Day 12 K/BxN serum-induced CXCR6^−/−^ mice (n = 10 sections from 10 mice for the Wt groups, and n = 10 sections from four mice in the CXCR6^−/−^ group (400×)).

Wt and CXCR6^−/−^ mice were induced with K/BxN serum, joints harvested and tissue sections immunostained for Id1. Day 12 Wt mice show clear expression of Id1 positive ECs, whereas CXCR6^−/−^ mice do not (400×, Figure 6A). The results are graphed and show that day 0 and 12 Wt mice have Id1 expressing EPCs in joint tissue, but Id1 positive cells were not detected in Day 12 K/BxN serum-induced CXCR6^−/−^ mice (Figure 6B).

## Discussion

Neovascularization occurs by one of two mechanisms: angiogenesis, the replication and reorganization of pre-existing microvascular ECs [25], or by vasculogenesis, the recruitment of EPCs that subsequently incorporate into the existent tissues and differentiate into mature functional ECs [26]. However, the lack of a single marker to unambiguously track EPCs has led to several recent studies failing to identify these cells in specific mouse tumor models [10,11,27,28]. As a result, it is argued that EPCs may not be a viable target for RA therapy as these cells have not been found in appreciable numbers in inflamed synovium. However, these same findings raised significant concerns as to whether the same EPC population is being truly monitored *in vivo*, and has imposed tremendous limitations on the assessment of the biological function of EPCs, as well as their potential use as a therapeutic strategy targeting neovasculature in RA tissues [29].

Notably, RA patients show increased numbers of circulating EPCs that correlate with Disease Activity Scores using 28 joint counts (DAS28), signifying that EPCs are likely elevated and recruited to inflamed tissues for the purposes of synovial vasculogenesis [30]. Additionally, growing evidence has suggested that EPCs contribute to the homeostasis of the physiologic vascular network [5], as well as contribute to vascular remodeling of RA synovium by recruiting BM derived circulating EPCs [20]. We believe that analysis of EPC mediated migration using Id1 as a selective and unique EPC marker may be an intriguing strategy for identifying and targeting EPC vascular integration during the course of active arthritis. Histologic analysis of ST revealed that Id1 is highly expressed in the vasculature of RA ST, but less so in OA or NL ST, suggesting that the micro-environment of the RA joint either facilitates Id1 expression and/or is favorable for EPC migration. We used fluorescence histology to examine the percentage of blood vessels containing EPCs by staining Id1, and found an elevated percentage of Id1 containing blood vessels in RA compared to OA and NL STs. These findings are in complete agreement with those of Sakurai *et al*., who showed substantial expression of Id1 and Id3 in RA compared to OA synovium at the protein and transcriptional levels [31].

One of the many interesting features of Id1 is its ability to not only inhibit genes related to cell maturity and growth, but to equally repress inhibitors of angiogenesis. Previous studies showed that Id1 regulates angiogenesis through transcriptional repression of thrombospondin-1 [32]. It was subsequently shown that Id1 can also repress p21 expression to control EPC growth and maturation in the BM. Because of the ability of Id1 to down-regulate expression of these potent repressors, it was reported that Id1 can function as an effective pro-angiogenic mediator produced by EPCs and pluripotent stem cells [33]. This idea was reinforced by reports identifying Id1 and Id3 as negative regulators of pluripotent stem cell maturation [34], and supported the notion that Id1 is uniquely expressed in progenitor cells. These findings also pointed to Id1 as a selective marker for progenitor cells that could be used to identify EPCs in tissues characterized by extensive vascular remodeling. Gao *et al.* subsequently showed that it was possible to identify, track and target BM-derived EPCs *in vivo* using a mouse model of pulmonary metastasis by way of Id1 expression. The authors went on to show that targeting EPCs in this way blocked EPC mobilization, caused angiogenesis inhibition, impaired the spread of metastasis, and increased the survival of tumor bearing mice [9,29].

We surmised that Id1 could also be used to identify EPCs in RA tissues, and examined if Id1 could be expressed and secreted as well as exhibit angiogenic activity after exiting the cell. We show that Id1 can be secreted, is highly expressed in RA SF, and can be correlated with CXCL16 expression. Indeed, approximately 56% of the variability of CXCL16 in RA SFs can be accounted for by Id1, which is relatively large considering the numerous angiogenic factors in the RA joint [35]. This indicates that CXCL16 (therefore, CXCR6 expressing cells) is linked with Id1 expression (therefore, EPCs) in RA tissues. We measured Id1 in RA SFs and compared this to the levels found in OA SFs as well as SFs from patients with other diseases. The OA SFs serve as non-inflammatory, non-autoimmune controls for the RA SFs. Although not ideal, we do not have access to NL SFs as these are not available. For this reason, we have used OA SFs for comparison of soluble pro-inflammatory mediators (for example, cytokines and chemokines) in many previous studies [14]. It should also be noted that the heterogeneity of the SFs from the “other disease” group was intended to show that the Id1 levels in OA SFs and SFs from a diverse patient population can be used together to verify that Id1 is uniquely elevated in RA SF, and can be correlated to RA SF CXCL16 expression.

Ling *et al.* previously reported that Id1 protein can be regulated by TNF-α in prostate cancer cell lines. They found that exposure to TNF-α in two different cell lines (DU145 and PC-3) resulted in a rapid and significant down-regulation of Id1 protein [36]. We show that Id1 mRNA transcripts can be detected in HMVECs and EPCs, and that CXCL16, but not TNF-α, can up-regulate Id1 expression in EPCs. It is important to point out that although we found Id1 mRNA in both HMVECs and EPCs, it was only actively transcribed in EPCs upon CXCL16 stimulation. Id1 mRNA expression in mature cells, such as HMVECs, is likely due to the remarkable stability of Id1 mRNA, over eight-fold higher than comparable mRNAs in induced pluripotent stem cells [37]. We also found that TNF-α destabilized Id1 mRNA in HMVECs, but not EPCs, consistent with previous reports [36]. This raises the possibility that TNF-α and CXCL16 activate specific mRNA binding proteins in ECs and EPCs, that may bind 3′ untranslated regions (UTRs) effecting Id1 mRNA stability, in a similar way to that showed previously with granulocyte macrophage-colony stimulating factor (GM-CSF) and ionophore in 3D10 cells [38]. It is tempting to speculate that as a progenitor cell begins to mature in the RA synovium, locally expressed cytokines, such as TNF-α and CXCL16, may affect Id1 stability and expression, thereby permitting the EPC to mature and incorporate into the existing vasculature in the RA joint.

We next examined the possibility that secreted Id1 could recruit HMVECs *in vitro*. Id1 effectively recruits HMVECs in a dose-dependent manner that can be inhibited by NFκB and PI_3_K signaling inhibitors. This demonstrates that mature ECs actively bind Id1 (indicating a receptor), and induce signaling pathways. HMVECs also respond to Id1 in a Matrigel tube forming assay. At the same concentrations, we observed chemotaxis (1 and 10 nM), HMVECs made extensive networks of tubes in response to Id1. In addition, diluted RA SF also had a similar effect on HMVECs in Matrigel, but was reversed with removal of Id1 in four out of five RA SFs examined. To further explore the possibility that Id1 was a potent mediator of vasculogenesis, we looked at its ability to recruit EPCs to RA ST in the SCID mouse chimera system. We show that Id1 is a potent recruitment factor for EPCs, and that RA SF depleted of Id1 lost approximately 50% of its EPC recruitment activity *in vivo*.

To date, stromal derived factor-1α (SDF-1α)/CXCL12 and its receptor CXCR4 have been acknowledged to be the primary ligand-receptor pair for EPC chemotactic activity. However, the expression of SDF-1/CXCL12 is comparatively much lower in RA SF to that of CXCL16 [14,39]. Interestingly, a Fluorescence Activated Cell Sorter (FACS/flow cytometry) study reported high percentages (96 ± 2%; mean ± SEM) of primary BM-derived murine MSCs expressing CXCR6, but not CXCR4, on their cell surface. Notably, CXCR6 and CXCR4 were equally expressed on a high proportion (95 ± 1%) of human BM derived MSCs [17]. With this in mind, we stained the joint tissues of Wt and CXCR6^−/−^ K/BxN serum-induced mice for Id1. We initially found that Day 0 Wt mice show low levels of EC staining for Id1, which was elevated by Day 12. However, Day 12 CXCR6^−/−^ mice had significantly reduced arthritis and vasculature (data not shown) and completely lacked EC staining for Id1, showing that Id1 and the CXCL16-CXCR6 ligand-receptor pathway are linked and work together to recruit EPCs from the BM to the synovium.

Before use in the K/BxN serum transfer arthritis studies, the CXCR6^−/−^ mice were backcrossed onto the C57BL/6 background for more than 10 generations, making the C57BL/6 mouse the natural control for these experiments. We found that healthy mice bred on the C57BL/6 background do not express appreciable amounts of Id1. We should also note that the expression of Id1 is generally very low in joint tissues in the K/BxN serum transfer arthritis model, apart from peak arthritis times (days 5 to 12). This is likely because it takes significant time for vasculature to develop in joint tissues of arthritic mice, even when using a potent acute model of arthritis like K/BxN. This is due in part because BM derived EPCs are required to migrate to the joints and become embedded into the developing microvasculature. This is the reasoning behind looking at Day 12 of arthritis induction (that is, close enough to peak arthritis but before the joint swelling/inflammation resolves).

We recently showed a correlation among CXCR6 expression, arthritis development and angiogenesis in mice using the K/BxN serum transfer model. We showed that deletion of CXCR6 (the only known receptor for CXCL16) prevents arthritis development, severity and joint tissue vascularity in mice in response to K/BxN serum [40]. Using the same mice and model, we now show that Id1 is markedly down-regulated in the joints of mice lacking CXCR6. In our previously published study, we demonstrated that the CXCR6-CXCL16 receptor-ligand pair is actively involved in recruiting EPCs to RA ST [40]. Because EPCs express CXCR6 and respond to CXCL16 *in vivo*, we hypothesized that Id1 expressing EPCs could be found in the neovasculature of inflamed joint tissue, and that this process could be inhibited by disruption of the CXCR6-CXCL16 receptor-ligand pair.

We believe that studies such as this will lead to a better understanding of the mechanisms that regulate EPC recruitment and differentiation into mature ECs. EPC recruitment processes that regulate neovascularization are relevant to diverse clinical scenarios, from inhibiting RA and tumor progression to replenishing the blood supply of ischemic hearts. In some of these disease states, such as myocardial damage, the favorable end goal is to find means to enhance the contribution of EPCs to new blood vessel formation, with the desired result of revitalizing the blood supply to damaged and imperfectly functioning tissues. In the case of tumorigenesis and RA, the opposite effect (that is, inhibiting or preventing EPC dependent vascular expansion), in an effort to starve the affected tissues and thus delay or reverse expansion of invasive tissues, is the preferred effect. With a clearer understanding of the biological underpinnings that guide EPCs to the microcirculatory beds of inflamed or angiogenic tissues, we might be able to take advantage of EPC homing in RA by targeting EPC chemokine receptors, such as CXCR6, or by using such cells as vehicles for the delivery of biotoxins or of gene therapy agents that have anti-inflammatory activity, cause neovessel obliteration, and/or suppress synovial proliferation. We show herein that CXCL16 and Id1 are linked and central to EPC recruitment in RA. We also show that Id1 can be secreted and induce angiogenic activity in mature ECs (HMVECs). This indicates that Id1 is not only self-regulatory in EPCs, but after secretion, can induce potent angiogenic responses.

## Conclusion

Our data indicate that Id1 can be secreted into the RA SF and correlate with CXCL16 expression. In addition, CXCR6^−/−^ arthritic mice have markedly reduced Id1 expression in the K/BxN serum transfer model of arthritis. We also found that Id1 is potently angiogenic, and can be up-regulated in HMVECs and EPCs by TNF-α and, especially, CXCL16. These results indicate that CXCL16 can be highly correlated with Id1 expression, and that Id1 is active in EPC recruitment and blood vessel formation in the RA joint.

## Abbreviations

bFGF: Basic fibroblast growth factor; BM: Bone marrow; DAB: 3,3′-diaminobenzidine; DAPI: 4′,6-diamidino-2-phenylindole; DNA: Deoxyribonucleic acid; ECs: Endothelial cells; ELISA: Enzyme-linked immunosorbant assay; EPCs: Endothelial progenitor cells; FBS: Fetal bovine serum; G-CSF: Granulocyte-colony stimulating factor; HLH: Helix-loop-helix; HMVECs: Human dermal microvascular endothelial cells; Id1: Inhibitor of DNA binding 1; IF: Immunofluorescence; mRNA: messenger RNA; MSCs: Mesenchymal stem cells; NFκB: Nuclear factor kappa B; NL: Normal; OA: osteoarthritis; PB: Peripheral blood; PBS: Phosphate buffered saline; qPCR: Quantitative polymerase chain reaction; RA: Rheumatoid arthritis; rhuId1: recombinant human Id1; RNA: Ribonucleic acid; SCID: Severe combined immunodeficient; (SDF-1α)/CXCL12: Stromal derived factor-1α; SF: Synovial fluid; ST: Synovial tissue; TNF-α: Tumor necrosis factor-α; vWF: von Willebrand factor; Wt: Wild type.

## Competing interests

The authors declare that they have no competing interests.

## Authors’ contributions

TI participated in research conception and design, data collection and analysis, critical revision, manuscript writing and final approval of the manuscript. MAA, ASA, CMH, GE and CKH III participated in data collection and analysis, critical revision and final approval of the manuscript. AEK contributed to manuscript writing, critical revision and final approval of the manuscript. JHR contributed to conception and design, data collection and analysis, critical revision, manuscript writing and final approval of the manuscript. All authors read and approved the final manuscript.

## References

[B1] Koch AE Angiogenesis as a target in rheumatoid arthritis Ann Rheum Dis 2003 62 ii60 ii67 14532152 10.1136/ard.62.suppl_2.ii60 PMC1766740

[B2] Patan S Vasculogenesis and angiogenesis as mechanisms of vascular network formation, growth and remodeling J Neurooncol 2000 50 1 15 10.1023/A:1006493130855 11245270

[B3] Gonzalez-Amaro R Sanchez-Madrid F Cell adhesion molecules: selectins and integrins Crit Rev Immunol 1999 19 389 429 10647744

[B4] Godessart N Kunkel SL Chemokines in autoimmune disease Curr Opin Immunol 2001 13 670 675 10.1016/S0952-7915(01)00277-1 11677088

[B5] Distler JH Beyer C Schett G Luscher TF Gay S Distler O Endothelial progenitor cells: novel players in the pathogenesis of rheumatic diseases Arthritis Rheum 2009 60 3168 3179 10.1002/art.24921 19877034

[B6] Qin S Rottman JB Myers P Kassam N Weinblatt M Loetscher M Koch AE Moser B Mackay CR The chemokine receptors CXCR3 and CCR5 mark subsets of T cells associated with certain inflammatory reactions J Clin Invest 1998 101 746 754 10.1172/JCI1422 9466968 PMC508621

[B7] Isner JM Kalka C Kawamoto A Asahara T Bone marrow as a source of endothelial cells for natural and iatrogenic vascular repair Ann N Y Acad Sci 2001 953 75 84 11795425 10.1111/j.1749-6632.2001.tb02075.x

[B8] Nolan DJ Ciarrocchi A Mellick AS Jaggi JS Bambino K Gupta S Heikamp E McDevitt MR Scheinberg DA Benezra R Mittal V Bone marrow-derived endothelial progenitor cells are a major determinant of nascent tumor neovascularization Genes Dev 2007 21 1546 1558 10.1101/gad.436307 17575055 PMC1891431

[B9] Gao D Nolan DJ Mellick AS Bambino K McDonnell K Mittal V Endothelial progenitor cells control the angiogenic switch in mouse lung metastasis Science 2008 319 195 198 10.1126/science.1150224 18187653

[B10] Bertolini F Shaked Y Mancuso P Kerbel RS The multifaceted circulating endothelial cell in cancer: towards marker and target identification Nat Rev Cancer 2006 6 835 845 10.1038/nrc1971 17036040

[B11] Shaked Y Ciarrocchi A Franco M Lee CR Man S Cheung AM Hicklin DJ Chaplin D Foster FS Benezra R Kerbel RS Therapy-induced acute recruitment of circulating endothelial progenitor cells to tumors Science 2006 313 1785 1787 10.1126/science.1127592 16990548

[B12] Perk J Gil-Bazo I Chin Y de Candia P Chen JJ Zhao Y Chao S Cheong W Ke Y Al-Ahmadie H Gerald WL Brogi E Benezra R Reassessment of id1 protein expression in human mammary, prostate, and bladder cancers using a monospecific rabbit monoclonal anti-id1 antibody Cancer Res 2006 66 10870 10877 10.1158/0008-5472.CAN-06-2643 17108123

[B13] Lyden D Young AZ Zagzag D Yan W Gerald W O’Reilly R Bader BL Hynes RO Zhuang Y Manova K Benezra R Id1 and Id3 are required for neurogenesis, angiogenesis and vascularization of tumour xenografts Nature 1999 401 670 677 10.1038/44334 10537105

[B14] Ruth JH Haas CS Park CC Amin MA Martinez RJ Haines GK 3rd Shahrara S Campbell PL Koch AE CXCL16-mediated cell recruitment to rheumatoid arthritis synovial tissue and murine lymph nodes is dependent upon the MAPK pathway Arthritis Rheum 2006 54 765 778 10.1002/art.21662 16508941 PMC1472704

[B15] Nanki T Shimaoka T Hayashida K Taniguchi K Yonehara S Miyasaka N Pathogenic role of the CXCL16-CXCR6 pathway in rheumatoid arthritis Arthritis Rheum 2005 52 3004 3014 10.1002/art.21301 16200580

[B16] van der Voort R van Lieshout AW Toonen LW Sloetjes AW van den Berg WB Figdor CG Radstake TR Adema GJ Elevated CXCL16 expression by synovial macrophages recruits memory T cells into rheumatoid joints Arthritis Rheum 2005 52 1381 1391 10.1002/art.21004 15880344

[B17] Chamberlain G Wright K Rot A Ashton B Middleton J Murine mesenchymal stem cells exhibit a restricted repertoire of functional chemokine receptors: comparison with human PLoS ONE 2008 3 e2934 10.1371/journal.pone.0002934 18698345 PMC2488395

[B18] Monach P Hattori K Huang H Hyatt E Morse J Nguyen L Ortiz-Lopez A Wu HJ Mathis D Benoist C The K/BxN mouse model of inflammatory arthritis: theory and practice Methods Mol Med 2007 136 269 282 10.1007/978-1-59745-402-5_20 17983155

[B19] Moore XL Lu J Sun L Zhu CJ Tan P Wong MC Endothelial progenitor cells’ “homing” specificity to brain tumors Gene Ther 2004 11 811 818 10.1038/sj.gt.3302151 15057261

[B20] Silverman MD Haas CS Rad AM Arbab AS Koch AE The role of vascular cell adhesion molecule 1/ very late activation antigen 4 in endothelial progenitor cell recruitment to rheumatoid arthritis synovium Arthritis Rheum 2007 56 1817 1826 10.1002/art.22706 17530710

[B21] Katschke KJ Jr Rottman JB Ruth JH Qin S Wu L LaRosa G Ponath P Park CC Pope RM Koch AE Differential expression of chemokine receptors on peripheral blood, synovial fluid, and synovial tissue monocytes/macrophages in rheumatoid arthritis Arthritis Rheum 2001 44 1022 1032 10.1002/1529-0131(200105)44:5<1022::AID-ANR181>3.0.CO;2-N 11352233

[B22] Ruth JH Rottman JB Katschke KJ Qin S Wu L LaRosa G Ponath P Pope RM Koch AE Selective lymphocyte chemokine receptor expression in the rheumatoid joint Arthritis Rheum 2001 44 2750 2760 10.1002/1529-0131(200112)44:12<2750::AID-ART462>3.0.CO;2-C 11762935

[B23] Everly DN Jr Mainou BA Raab-Traub N Induction of Id1 and Id3 by latent membrane protein 1 of Epstein-Barr virus and regulation of p27/Kip and cyclin-dependent kinase 2 in rodent fibroblast transformation J Virol 2004 78 13470 13478 10.1128/JVI.78.24.13470-13478.2004 15564458 PMC533955

[B24] Marotte H Ahmed S Ruth JH Koch AE Blocking ERK-1/2 reduces tumor necrosis factor alpha-induced interleukin-18 bioactivity in rheumatoid arthritis synovial fibroblasts by induction of interleukin-18 binding protein A Arthritis Rheum 2010 62 722 731 10.1002/art.27269 20131228 PMC2855552

[B25] Folkman J Haudenschild C Angiogenesis by capillary endothelial cells in culture Trans Ophthalmol Soc U K 1980 100 346 353 6171066

[B26] Masuda H Asahara T Post-natal endothelial progenitor cells for neovascularization in tissue regeneration Cardiovasc Res 2003 58 390 398 10.1016/S0008-6363(02)00785-X 12757873

[B27] Gao D Mittal V The role of bone-marrow-derived cells in tumor growth, metastasis initiation and progression Trends Mol Med 2009 15 333 343 10.1016/j.molmed.2009.06.006 19665928

[B28] Purhonen S Palm J Rossi D Kaskenpaa N Rajantie I Yla-Herttuala S Alitalo K Weissman IL Salven P Bone marrow-derived circulating endothelial precursors do not contribute to vascular endothelium and are not needed for tumor growth Proc Natl Acad Sci U S A 2008 105 6620 6625 10.1073/pnas.0710516105 18443294 PMC2365563

[B29] Mellick AS Plummer PN Nolan DJ Gao D Bambino K Hahn M Catena R Turner V McDonnell K Benezra R Brink R Swarbrick A Mittal V Using the transcription factor inhibitor of DNA binding 1 to selectively target endothelial progenitor cells offers novel strategies to inhibit tumor angiogenesis and growth Cancer Res 2010 70 7273 7282 10.1158/0008-5472.CAN-10-1142 20807818 PMC3058751

[B30] Jodon de Villeroche V Avouac J Ponceau A Ruiz B Kahan A Boileau C Uzan G Allanore Y Enhanced late-outgrowth circulating endothelial progenitor cell levels in rheumatoid arthritis and correlation with disease activity Arthritis Res Ther 2010 12 R27 10.1186/ar2934 20158894 PMC2875661

[B31] Sakurai D Yamaguchi A Tsuchiya N Yamamoto K Tokunaga K Expression of ID family genes in the synovia from patients with rheumatoid arthritis Biochem Biophys Res Commun 2001 284 436 442 10.1006/bbrc.2001.4974 11394898

[B32] Volpert OV Pili R Sikder HA Nelius T Zaichuk T Morris C Shiflett CB Devlin MK Conant K Alani RM Id1 regulates angiogenesis through transcriptional repression of thrombospondin-1 Cancer Cell 2002 2 473 483 10.1016/S1535-6108(02)00209-X 12498716

[B33] Ciarrocchi A Jankovic V Shaked Y Nolan DJ Mittal V Kerbel RS Nimer SD Benezra R Id1 restrains p21 expression to control endothelial progenitor cell formation PLoS ONE 2007 2 e1338 10.1371/journal.pone.0001338 18092003 PMC2129121

[B34] Hong SH Lee JH Lee JB Ji J Bhatia M ID1 and ID3 represent conserved negative regulators of human embryonic and induced pluripotent stem cell hematopoiesis J Cell Sci 2011 124 1445 1452 10.1242/jcs.077511 21486943

[B35] Szekanecz Z Koch AE Angiogenesis and its targeting in rheumatoid arthritis Vascul Pharmacol 2009 51 1 7 10.1016/j.vph.2009.02.002 19217946 PMC2917972

[B36] Ling MT Kwok WK Fung MK Xianghong W Wong YC Proteasome mediated degradation of Id-1 is associated with TNFalpha-induced apoptosis in prostate cancer cells Carcinogenesis 2006 27 205 215 10.1093/carcin/bgi217 16123120

[B37] Neff AT Lee JY Wilusz J Tian B Wilusz CJ Global analysis reveals multiple pathways for unique regulation of mRNA decay in induced pluripotent stem cells Genome Res 2012 22 1457 1467 10.1101/gr.134312.111 22534399 PMC3409259

[B38] Ruth JH Esnault S Jarzembowski JA Malter JS Calcium ionophore upregulation of AUUUA-specific binding protein activity is contemporaneous with granulocyte macrophage colony-stimulating factor messenger RNA stabilization in AML14.3D10 cells Am J Respir Cell Mol Biol 1999 21 621 628 10.1165/ajrcmb.21.5.3694 10536121

[B39] Kim KW Cho ML Kim HR Ju JH Park MK Oh HJ Kim JS Park SH Lee SH Kim HY Up-regulation of stromal cell-derived factor 1 (CXCL12) production in rheumatoid synovial fibroblasts through interactions with T lymphocytes: role of interleukin-17 and CD40L-CD40 interaction Arthritis Rheum 2007 56 1076 1086 10.1002/art.22439 17393416

[B40] Isozaki T Arbab AS Haas CS Amin MA Arendt MD Koch AE Ruth JH Evidence that CXCL16 is a potent mediator of angiogenesis and is involved in endothelial progenitor cell chemotaxis: studies in mice with K/BxN serum-induced arthritis Arthritis Rheum 2013 65 1736 1746 10.1002/art.37981 23633118 PMC3701743

